# Rifabutin Is Active against Mycobacterium abscessus in Mice

**DOI:** 10.1128/AAC.01943-19

**Published:** 2020-01-27

**Authors:** Thomas Dick, Sung Jae Shin, Won-Jung Koh, Véronique Dartois, Martin Gengenbacher

**Affiliations:** a Center for Discovery and Innovation, Hackensack Meridian Health, Nutley, New Jersey, USA; b Department of Medical Sciences, Hackensack Meridian School of Medicine at Seton Hall University, Nutley, New Jersey, USA; c Department of Microbiology, Institute for Immunology and Immunological Diseases, Brain Korea 21 PLUS Project for Medical Science, Yonsei University College of Medicine, Seoul, South Korea; d Division of Pulmonary and Critical Care Medicine, Department of Medicine, Samsung Medical Center, Sungkyunkwan University School of Medicine, Seoul, South Korea; e Department of Medicine, New Jersey Medical School, Rutgers, The State University of New Jersey, Newark, New Jersey, USA

**Keywords:** nontuberculous mycobacteria, NTM, rifamycin, mouse model, animal model

## Abstract

There is no reliable cure for Mycobacterium abscessus lung disease. Rifampin is not used clinically due to poor *in vitro* potency. In contrast, we have shown that rifabutin, another approved rifamycin used to treat tuberculosis, is potent *in vitro* against M. abscessus. Here, we report that rifabutin is as active as clarithromycin against M. abscessus K21 in NOD.CB17-Prkdc^scid^/NCrCrl mice. This suggests that rifabutin should be considered a repurposing candidate for patients with M. abscessus disease.

## TEXT

Nontuberculous mycobacterium (NTM) infections are becoming increasingly prevalent and surpass tuberculosis in many developed countries, including the United States (1). The majority of NTM disease is caused by the Mycobacterium avium complex and Mycobacterium abscessus (2). M. abscessus is intrinsically resistant to many drug classes, rendering the development of new antibiotics extremely challenging (3–5). Certain lung conditions, including cystic fibrosis and chronic obstructive pulmonary disease, increase the likelihood of developing NTM lung disease (6).

Repurposing existing antibiotics is a rapid way forward to address the urgent medical needs related to the rise of NTM infections (7). By screening a library of approved pharmaceuticals for growth inhibition of M. abscessus, we recently identified rifabutin (RFB) as a potential repurposing candidate (8). This RNA polymerase-targeting antibiotic is currently in clinical use for the treatment of tuberculosis and infections caused by M. avium complex (9, 10). Interestingly, other rifamycins, including rifampin (RIF) and rifapentine, are only poorly active against M. abscessus (8). Our previous work demonstrated that RFB not only inhibits growth but is also bactericidal against all three M. abscessus subspecies, i.e., M. abscessus subsp. *abscessus*, M. abscessus subsp. *bolletii*, and M. abscessus subsp. *massiliense* (8).

Motivated by these findings, the current work evaluates RFB in a murine model of NTM infection. Most mouse strains are highly resistant to M. abscessus infection and eventually clear the pathogen (11). However, mice with certain immune defects, including severe combined immunodeficient (SCID), interferon-γ knockout, and granulocyte-macrophage colony-stimulating factor knockout mice, develop a productive infection followed by a chronic phase with sustained bacterial burden in organs upon systemic inoculation with a high dose of M. abscessus (11, 12). We used 8-week-old female NOD.CB17-Prkdc^scid^/NCrCrl mice (NOD SCID; Charles River Laboratories), which have impaired B and T lymphocytes and deficient natural killer cell function. To achieve direct pulmonary infection, ∼10^6^ CFU of M. abscessus subsp. *abscessus* K21 isolated from a patient at the Samsung Medical Center, Seoul, South Korea, were intranasally delivered to anesthetized mice. M. abscessus K21 shows a rough colony morphotype when grown on Middlebrook 7H11 agar, harbors the C28 sequevar of *erm*(41), and is thus macrolide sensitive. All experiments involving live animals were approved by the Center for Discovery and Innovation, Institutional Animal Care and Use Committee.

**FIG 1:**
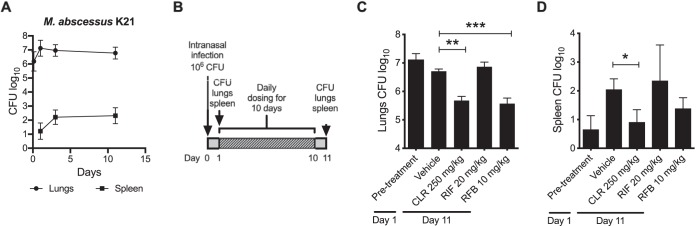
Rifabutin kills M. abscessus in mice. (A) Kinetics of bacterial burden in lungs and spleen of M. abscessus K21-infected NOD.CB17-Prkdc^scid^/NCrCrl mice. The inoculum of 10^6^ CFU was delivered intranasally. At designated time points, lungs and spleens of 4 animals were homogenized and plated on agar for CFU determination. (B) Schematic representation of the murine M. abscessus lung infection model used in this study. (C and D) Animals infected with M. abscessus K21 underwent drug treatment for 10 consecutive days. Drugs were administered once daily by oral gavage to groups of 6 mice per study group. At 11 days postinfection, organ homogenates were plated on agar to determine the bacterial load. Results were analyzed using one-way analysis of variance (ANOVA) multicomparison and Tukey’s posttest. *, *P < *0.05; **, *P < *0.01; ***, *P < *0.001. MICs reducing growth of M. abscessus K21 by 90% over 3 days for clarithromycin (CLR), rifampin (RIF), and rifabutin (RFB) were 0.6 μM, 50 μM, and 2.4 μM, respectively. CFU kinetics was carried out twice, and the drug efficacy study was done three times. Representative data sets are shown.

First, we characterized the kinetics of M. abscessus K21 infection in NOD SCID mice. At designated time points postinfection, groups of 4 mice were euthanized, and the bacterial burden in lungs and spleens was assessed by plating serial dilutions of organ homogenates on Middlebrook 7H11 agar supplemented with 10% oleic acid-albumin-dextrose-catalase and 0.2% glycerol. Colonies were counted after 5 days of incubation at 37°C. Results show that the lung bacterial burden increased 10-fold within the first day of infection and stabilized at roughly 10^7^ CFU thereafter (Fig. 1A). CFU kinetics in spleens followed a similar profile but at a lower burden not exceeding 0.5 × 10^3^ CFU by day 11 postinfection (Fig. 1A). Thus, in NOD SCID mice, M. abscessus K21 produces a disease pattern consisting of a short 1-day acute phase followed by a chronic infection. To measure drug efficacy, we designed an infection model in which treatment is initiated 1 day postinfection for 10 consecutive days, at which point reduction of CFU in lungs and spleens is compared to nontreated controls (Fig. 1B).

Next, the efficacy of clarithromycin (CLR), RIF, and RFB was evaluated in NOD SCID mice at doses selected to reproduce the area under the curve of the plasma concentration-time profile seen in patients taking clinically approved doses of 600 mg RIF, 300 mg RBT, and 250 to 500 mg CLR twice daily (13). Delivery of the M. abscessus K21 intranasal inoculum in NOD SCID mice was verified 3 h postinfection by plating the lung homogenates of 4 mice on Middlebrook 7H11 agar. On day 1 postinfection, pretreatment bacterial loads were measured in 6 mice, and groups of 6 mice were randomly assigned to the drug treatment or control arm. Study drugs formulated in 0.5% carboxymethyl cellulose/0.5% Tween 80/sterile water or vehicle were administered by oral gavage once daily for 10 consecutive days. Twenty-four hours after the last dose (11 days postinfection), all mice were euthanized to assess the load of M. abscessus K21 in their lungs and spleen. The efficacy of a drug was defined as a statistically significant reduction of CFU in a study group relative to the vehicle control at the end of the experiment. The macrolide CLR served as a positive control and significantly reduced the bacterial load in lungs by 1 log at 250 mg/kg. RFB at 10 mg/kg showed efficacy similar to CLR, while RIF at 20 mg/kg had no effect, as expected given its poor *in vitro* potency against M. abscessus (Fig. 1C). Spleen CFU reduction generally followed the trend observed in lungs (Fig. 1D). Thus, RFB is bactericidal in a preclinical mouse model of M. abscessus lung infection.

The mechanism underlying RFB’s superior *in vitro* activity compared to RIF remains to be determined. RIF has been shown to be metabolized by M. abscessus (14). Additional mechanisms, including bacterial oxidation or efflux, may contribute to the intrinsic resistance of M. abscessus to RIF (15). Recent *in vitro* drug-drug potency interaction studies indicate that RFB synergizes with several frontline drugs used against M. abscessus disease and does not antagonize any NTM antibiotics (reviewed in reference 15). This suggests that RFB could be integrated into existing drug regimens. In addition, RFB has a favorable pharmacological profile (16, 17), shows much reduced CYP3A4 induction compared to RIF (18), and is bactericidal against intracellular M. abscessus (19). Collectively, these and our findings suggest that RFB should be considered a repurposing candidate for the treatment of patients with M. abscessus disease.
